# Supplementary material for: Targeted and Untargeted Amine Metabolite Quantitation in Single Cells with Isobaric Multiplexing
Source: Chemistry. 2024 Nov 10;30(72):e202403278. doi: 10.1002/chem.202403278 (PMC11665497; doi:10.1002/chem.202403278)
Supplement: Supplementary file 1 — Supporting Information [file CHEM-30-e202403278-s001.pdf]

Chemistry–A European Journal

Supporting Information

Targeted and Untargeted Amine Metabolite Quantitation in Single Cells with Isobaric Multiplexing

Juho Heininen, Parisa Movahedi, Tapio Kotiaho, Risto Kostiainen, Tapio Pahikkala, and Jaakko Teppo*

Supporting Information

Targeted and Untargeted Amine Metabolite Quantitation in Single Cells with Isobaric Multiplexing

Juho Heininen [1], Parisa Movahedi [2], Tapio Kotiaho [1, 3], Risto Kostiainen [1], Tapio Pahikkala [2], Jaakko Teppo [1]*

1 Drug Research Program and Division of Pharmaceutical Chemistry and Technology, Faculty of Pharmacy, P.O. Box 56, FI-00014 University of Helsinki, Finland

2 Department of Computing, Turku University, 20014, Turku, Finland

3 Department of Chemistry, Faculty of Science, P.O. Box 55, FI-00014 University of Helsinki, Finland

Contents

Materials and methods 3

Standards, cells, and reagents 3

Single cell sample preparation... 3

Validation and optimization sample preparation 4

LC-MS1 and LC-MS2 analysis 5

Data processing 6

Table S1. Labeled analytes and internal standards. 8

Table S2. TMT exact masses and reporter ions.. 9

Table S3. MS2 transition windows and precursors of the targeted tPRM method............................. 10

Table S4. Exclusion mass list. 11

Table S5. mzMine batch process. 12

Figure S1. Calibration Graphs 13

Figure S2. Retention time comparison on nLC and UHPLC. 14

Figure S3. Heatmaps of single cells. 15

Figure S4. PCA of full data and RLS selection 16

Figure S5. Example of MS2 scan matching for glutamic acid annotation.. 17

Materials and methods

Standards, cells, and reagents

Reagents. Amino acid standards and the corresponding amino acids with heavy ^{15}N and ^{13}C isotopes (Cambridge Isotope Laboratories) were used as internal standards (Table S1). These are referred to as light and heavy amino acid standard, respectively. LC-MS grade water and acetonitrile, both with 0.1% formic acid, were from Thermo Scientific. TMT0 and TMT10plex isobaric reagents, 1 M triethyl ammonium bicarbonate (TEAB) buffer, and 50% hydroxylamine solution were purchased from Thermo Scientific. Anhydrous dimethyl sulfoxide (DMSO) was acquired from Honeywell.

Cell culture, imaging, sorting, and lysis. TYK-nu and TYK-nu.CP-r cell lines were a kind gift from Jukka Saarinen, and the HEK293 cell line was received from Leena Pietilä (both from University of Helsinki). All cell lines tested negative for mycoplasma at the beginning of cell culture. The cells were grown at 37 °C under 5% CO_2 in antibiotic-free low glucose Dulbecco's Modified Eagle Medium with L-glutamine, pyruvate, and HEPES (Gibco, REF 22320022), supplemented with 10% fetal bovine serum (Gibco, REF 10270106). At >70% confluence, the cells were washed with phosphate-buffered saline (PBS; Gibco, REF 14200-067, diluted into 1X), detached using TrypLE Express (Gibco, REF 12604021), washed twice with cold PBS, and resuspended in cold PBS. The cells were kept on ice until sorting on the same day. Single cells were individually imaged and sorted using the CellenONE platform into 384-well plate wells containing 5 µL of water. Circularity, diameter, and elongation cut-off values were used to exclude doublets and debris. Sorted and imaged cells were frozen in -80 °C until cell lysis. During sorting, the cell viability was >85%, as assessed with LUNA-FL automated cell counter. For the samples prepared in bulk, cells were sorted with flow cytometry using BD FACSAria II. Cell lysis was done one plate at a time by a freeze-thaw lysis approach modified from that presented by Végvári *et al.* [1]. Cell lysis consisted of four 2-min cycles of freezing in liquid nitrogen, with thawing in 37°C between the freezing cycles. Next, the cells were sonicated for 5 min and heated to 90 °C for 10 min. Finally, the lysates were centrifuged to the bottoms of the wells, and the 384-well plates were sealed using well plate sealing foil.

Single cell sample preparation

Multiplexed samples consisted of approximately 120 cell-equivalent (120 fmol compared to approx. 1 fmol amount in cells) carrier (labeled with TMT10plex label 126) of either analyte standard for targeted approach or 400 cell equivalent cell lysate for untargeted approach, blank (127N), blank with internal standards of heavy amino acids (IS-blank, 128N), and six single cells (128C, 129N, 129C, 130N, 130C, and 131; exact masses of reporter ions in Table S2). TMT10plex label 127C was not used due to isotopic interference from the carrier, caused by isotopic impurity in the labeling reagents. The labeling principle is the same as previously reported.[2] DMSO was used as substitute for acetonitrile to limit solvent evaporation and obtain robust low microliter dispensing.[3]

Carrier for targeted metabolomics was prepared by mixing 79 µL of 100 mM TEAB, 180 µL of a solution containing 240 nM of each light amino acid standard in water, and 4.5 µL of 960 nM heavy amino acid standard in water, in a low-binding Eppendorf tube. This was labelled using 12 µL of 17.2 mM TMT10plex 126 label

reagent dissolved in anhydrous DMSO, and allowed to react for one hour. Subsequently, the labelling reaction was quenched with 12 µL of 1% hydroxylamine, and this mixture was incubated for 15 minutes.

Carrier for untargeted metabolomics was prepared similarly as for targeted metabolomics, but using 180 µL of 80 cells/µL lysate in water instead of the light amino acid standard.

Blank was prepared using 260 µL of TEAB, labeled with 127N label reagent as described above. **IS blank** was produced by mixing 353 µL of 100 mM TEAB with 6 µL of 960 nM heavy amino acid standard, and labeled with 128N label reagent as described above.

The lysed **individual real single cell samples** on the 384-well plates were prepared using a Biomek i7 pipetting robot. 2 µL of 60 nM heavy amino acid standard (internal standard) was added into each well containing a lysed single cell, followed by labeling with 1 µL of one of the TMT10plex reagents (128C, 129N, 129C, 130N, 130C, or 131). To limit batch effects, label swap was carried out, so that the same cell line was not always labeled using same reagent. Labeling was continued for 1h in RT, and the labeling was then quenched using 1 µL of 1% hydroxylamine.

60 lysed and labeled individual single cells were used for targeted and 48 for untargeted metabolomics. Cell samples were combined in sets of six individual cells (labeled with one of each reagent from 128C to 131), along with 8 µL of the corresponding carrier, blank, and IS-blank into low binding 250 µL glass vial inserts. The samples were evaporated to dryness with a vacuum centrifuge and frozen to -80 °C until measurement. Prior to analysis, the samples were thawed and dissolved in 20 µL of eluent A used in nLC.

Validation and optimization sample preparation

MS2 sensitivity specific parameters were optimized using a synthetic single cell TMT10plex labeled sample in larger volume providing comparable measurements. This synthetic single cell sample consisted, similarly to real single cell samples, of carrier, blank, IS-blank and six single cell equivalent samples. Absolute quantitation was obtained using validation samples for calibration, linearity, LOD, LOQ, and precision.

Synthetic single cell sample, used for optimizing MS2 sensitivity specific parameters, was prepared so that one LC-MS injection of it corresponds to the analyte amount in one multiplexed single cell sample. 40 µl of 100 mM TEAB, 10 µl of 6 nM light amino acid standard, and 40 µL of 60 nM heavy amino acid standard were added into six separate low-binding Eppendorf tubes. Each of these was labeled with one TMT10plex isotopomer (128C-131, 17.2 mM in DMSO) for 1h and quenched with 20 µL of 1% hydroxylamine for 15 min. After labeling, the six synthetic single cell samples were combined with 100 µL of carrier for targeted metabolomics, 100 µL of blank, and 100 µL of IS-blank sample, and evaporated to dryness. The sample was then dissolved in 200 µL of eluent A.

TMT0-labeled sample, used for optimizing the general LC and MS parameters, was prepared in a low-binding Eppendorf tube by combining 120 µL of 100 mM TEAB, 750µL of 240 nM light amino acid standard, and 300 µL of 204 nM heavy amino acid standard. This was labeled with 60 µL of 17.2 mM of TMT0 in DMSO for 1h,

and quenched with 60 µL of 1% hydroxylamine. The sample was evaporated to dryness and dissolved into 600 µL of eluent A, and further diluted 1:1, 1:10 and 1:100 with eluent A.

Validation samples followed the same multiplexing framework, and they were prepared individually from stock solutions into 384-well plates using the Biomek i7 pipetting robot. For each sample, 10 µL of TEAB, 4 µL of 60 nM heavy amino acid standard, and varying amounts of light amino acid standard were added to obtain final sample amounts from 0.03 fmol to 120 fmol of light amino acids per sample injection. These samples were used for calibration, linearity, LOD, and LOQ, as well as multiple 0.3 fmol and 3.0 fmol sample amounts for precision (method repeatability). Samples were labeled with the appropriate TMT10plex isotopomer for 1h and quenched with 2 µL of 1% hydroxylamine for 15 minutes. The individual samples were then multiplexed and dried using a vacuum centrifuge. The validation samples were dissolved in 40 µL of eluent A before measurement.

LC-MS1 and LC-MS2 analysis

The LC-MS measurements were carried out using an Orbitrap Fusion tribrid mass spectrometer coupled to an EASY nLC 1200 liquid chromatograph with a nanospray Flex ion source (all from Thermo Fisher Scientific).

Chromatography was performed using Waters Acquity M class C-18 column (M-Class HSS T3 1.8 µm 75 µm x 200 mm) at room temperature, and the column was connected to a stainless steel nano bore emitter (30 µm I.D. 40 mm, Thermo Scientific) using a MicroTight union (PEEK 1/32” P-771, IDEX Health & Science). Eluent A consisted of 0.1% formic acid in water, and eluent B consisted of 0.1% formic and 80% acetonitrile in water. The flow rate was 300 nL min^{-1}. The gradient started from 1% eluent B and was then increased to 20% B in 20 min, to 95% B in the next 10 min, and held at 95% B for 20 min. The gradient was then decreased to 1% B in 1 min and held at 1% B for 9 minutes. The resulting total gradient time was 60 min. After each injection, the autosampler was washed with 28 µL of eluent A, 28 µL of eluent B, and again with 28 µL of eluent A.

Targeted tPRM windows were built with MS1 measurements of optimization samples using orbitrap with 120k mass resolution, *m/z* 100-1600 scan range, and 100 ms maximum injection time with 250% normalized AGC target. RF lens was optimized to 80%, spray voltage to 2.1 kV, sweep gas flow to 0.5 arbitrary units, and ion transfer tube temperature to 250 °C. The sweep cone was not installed, and the low-flow pure nitrogen sweep gas (not directed at the spray) was mainly used to keep contaminants in the room air from entering the open ion source and to stabilize the electrospray. The targeted MS2 measurements were performed using timed parallel reaction monitoring (tPRM) and timed precursor isolation based on analyte retention times measured with the MS1 method (tPRM windows in Table S3). Internal mass calibration with Easy-IC (fluoranthene) was used in the beginning of each measurement (RunStart EasyIC). MS2 precursor isolation was done using quadrupole mass window of 1.0 Da, and the higher-energy collisional dissociation (HCD) normalized collision energy was optimized to 35 % and used for all analytes. Reporter product ions were detected with a mass resolution of 60 000 and automatic scan range with defined low mass *m/z* 110 to ensure the detection of TMT reporter ions. AGC target was optimized to 250% with a maximum injection time of 150 ms.

For untargeted amine metabolomics, the analysis was done using data dependent acquisition (DDA) approach. The chromatographic gradient was adjusted to achieve 40% B instead of 20% at 20 min, but all other LC

parameters were kept similar as with the tPRM method. Spray voltage was set to 2.0 kV, sweep gas at 0.5 arbitrary unit flow and ion transfer tube to 250 °C. Internal mass calibration was done using RunStart Easy IC. MS scan (survey scan) consisted of orbitrap analysis with scan range *m/z* 100-1600, 120k mass resolution and with wide quadrupole isolation enabled. The maximum injection time was set to 50 ms with normalized AGC target of 250%. RF lens was set to 80%. The data dependent MS2 scans were based on top 7 precursors with targeted exclusion mass list (Table S4) of background ions determined based on a blank injection, intensity threshold of 3E4, and 20 s dynamic exclusion with 10 ppm mass tolerance. Also, apex detection filter of 4 s FWHM (based on MS1 optimization samples) and desired window of 40% was used. MS2 scans were done using orbitrap with 60k mass resolution and 1 Da isolation window. Scan range was set to begin from 110 Da to include the TMT reporter ions, and HCD fragmentation with 37% normalized collision energy was used. Maximum ion injection time was set to 118 ms with normalized AGC target to 250%.

Data processing

Raw and metadata are provided in MassIVE repository with identifier MSV000095047. For targeted metabolite analysis, raw tPRM MS2 data was imported to Skyline[4] (22.2.1.425), along with the analyte to reporter ion transition list. Peaks in the MS2 reporter ion chromatograms were integrated, and the results were exported to Rstudio for further analysis (Rstudio[5] version 2023.09.1 Build 494 with R[6] version 4.2.2). In R, data processing and visualization were done using the Cran package "tidyverse"[7] including "dplyr" and "tidyr". Raw mzML data was processed with "MSnbase"[8], and the graph zoom visualization was implemented using "ggforce"[9] and "scales"[10] and the color scheme was controlled using "RColorBrewer"[11].. Briefly, isobaric interference between reporter ion signals was corrected using inverse matrix multiplication and the isotopic purity of the label reagents provided in the Certificate of Analysis of the TMT reagent batch.

The targeted method was partially validated according to ICH guidelines[12] in terms of specificity, linearity, limit of quantitation (LOQ), and method repeatability. LOQ was determined as the lowest measured concentration producing a signal to noise ratio (S/N) > 10. Because MS2 noise within 5 ppm of TMT fragment ions is mainly not present, LOQ was further defined to have a relative residual between calibration line and actual measurement value below 60%. Analyte signals corrected for isotopic interference were calculated as relative to the corresponding corrected internal standard signals, and the absolute analyte amount in sample was calculated using calibration line with calibration sample concentrations. The calibration lines were produced using weighed (1/concentration) linear regression, and they were evaluated by R^2 as well as residuals of measurements against calibration line. Within range outliers were determined based on the residuals using Grubbs outlier test based on both residuals and relative residuals. Calibration sample 0.3 fmol was outlier with all analytes and 0.6 fmol with some analytes. Seven technical replicates at 0.3 fmol and 3 fmol per injection were measured for method repeatability. One technical replicate of 0.3 fmol was also tested to be an outlier and removed from all analytes.

The absolute sample amounts in single cells were calculated similarly using the calibration line equations. Cell volumes were calculated as spheres using diameter provided by CellenONE that was corrected for the platform systematic bias (7µm+10%) and the measured absolute sample amounts were divided by the cell volume to obtain cellular analyte concentration. The homoscedasticity of cellular concentrations was tested with Levene's test for

equal variances and Kruskal-Wallis one-way analysis of variance was used to compare the different analytes between cell types, followed by Dunn's post-hoc test.

For the untargeted amine metabolomics, Thermo raw data was converted to mzML using MSconvert[13] (3.0.22238) and imported to MZmine[14] 3 (version 3.9.0). The files were batch processed to feature table of features including MS2 scan (MzMine batch process is in Table S5) Data processing and analysis were performed using Python 3.11.3 in Visual Studio Code (version 1.89.1). The data was processed as data frame using the Pandas library, with numerical operations facilitated by NumPy[15]. Statistical computations, including standardization and normalization, cosine similarity and dimensionality reduction utilized functions from the SciPy[16] and scikit-learn[17] libraries. Batch effects were corrected using PyComBat[18], a Python implementation of the ComBat[19] algorithm. Visualization of the processed data was achieved through the use of Matplotlib[20] and Seaborn[21].

The absolute TMT10plex reporter ion intensities were parsed from MS2 scans and normalized to the carrier reporter ion intensity. Features were filtered to those detected in at least 25% of the cells, and accurate mass over 250 Da. To take into account the nLC retention time differences between samples, features within 0.005 Da and 0.4 min were binned, and features within bins were filtered to most abundant. Labeling byproducts were removed and the missingness was filled the smallest intensity, and the data was log2-transformed and Z-score standardized. Batch effects between multiplexed samples were corrected using ComBat, and the measurements were projected to their principal components PC1 and PC2.

The features driving the separation on PCA were inspected using supervised classification Greedy forward feature selection with regularized least squares (RLS) from Rlscore Library[22]. Greedy RLS initiates with an empty feature set, and during each iteration it selects and adds the feature that yields the best performance based on Leave-One-Out Cross-Validation (LOOCV). To prevent selection bias, the effectiveness of the feature selection method was assessed using the standard nested cross-validation technique where the feature selection was performed independently during each iteration of the outer cross-validation. The average performance of these models was then determined based on their one-vs-all prediction accuracy on the out-of-sample data across all rounds of the outer cross-validation. As there are three replications of each sampled cell, to count for the dependencies in sampling, leave-cell-out is used in the outer-loop.

The resulting features were searched against MS2 fragment spectra of TMT10plex labeled amines from our previous work[2] as well as MS1 against HMDB[23] library (downloaded in March 2024) filtered to amine containing chemical formulas with 5 mDa window. Feature MS2 annotation was considered also with MS2 spectra in open libraries in GNPS[24] with added TMT10plex modification (+229.162932 Da). Features were annotated using 5 level confidence hierarchy[25] to Level 2 using in-house high mass resolution MS2 data of TMT10plex labeled analytes and to Level 4 using the accurate mass precursors against nitrogen containing compounds of HMDB.

Table S1. Labeled analytes and internal standards.

Analytes, their measured ions, exact masses of ions as well as internal standard and internal standard exact mass.

**Amino acid**	**Ion**	**Exact mass**	**Internal standard**	**Exact mass**
Alanine	$[1M+TMT10+H]^+$	319.217893	Alanine ($^{13}C_3$ $^{15}N_1$)	323.224983
Arginine	$[1M+TMT10+H]^+$	404.281893	Arginine ($^{13}C_6$ $^{15}N_4$)	414.290153
Aspartic acid	$[1M+TMT10+H]^+$	363.207723	Aspartic acid ($^{13}C_4$ $^{15}N_1$)	368.218163
Glutamic acid	$[1M+TMT10+H]^+$	377.223373	Glutamic acid ($^{13}C_5$ $^{15}N_1$)	383.237163
Glycine	$[1M+TMT10+H]^+$	305.202243	Glycine ($^{13}C_2$ $^{15}N_1$)	308.205983
Histidine	$[1M+TMT10+H]^+$	385.239693	Histidine ($^{13}C_6$ $^{15}N_3$)	394.250913
Isoleucine	$[1M+TMT10+H]^+$	361.264843	Isoleucine ($^{13}C_6$ $^{15}N_1$)	368.281983
Leucine	$[1M+TMT10+H]^+$	361.264843	Leucine ($^{13}C_6$ $^{15}N_1$)	368.281983
Lysine**	$[1M+2TMT10+2H]^{2+}$	303.222973	Lysine ($^{13}C_6$ $^{15}N_2$)	307.230063
Methionine	$[1M+TMT10+H]^+$	379.221263	Methionine ($^{13}C_5$ $^{15}N_1$)	385.235053
Phenylalanine	$[1M+TMT10+H]^+$	395.249193	Phenylalanine ($^{13}C_9$ $^{15}N_1$)	405.276383
Proline	$[1M+TMT10+H]^+$	345.233543	Proline ($^{13}C_5$ $^{15}N_1$)	351.247333
Serine	$[1M+TMT10+H]^+$	335.212803	Serine ($^{13}C_3$ $^{15}N_1$)	339.219893
Threonine	$[1M+TMT10+H]^+$	349.228453	Threonine ($^{13}C_4$ $^{15}N_1$)	354.238893
Tyrosine	$[1M+TMT10+H]^+$	411.244103	Tyrosine ($^{13}C_9$ $^{15}N_1$)	421.271293
Valine	$[1M+TMT10+H]^+$	347.249193	Valine ($^{13}C_5$ $^{15}N_1$)	353.262983
Cystine**	$[1M+2TMT10+2H]^{2+}$	350.182133	Cystine ($^{13}C_6$ $^{15}N_2$)	354.189223

** Lysine and cystine are double labeled and doubly charged

Table S2. TMT exact masses and reporter ions.

Labeling exact mass change with TMT10plex reagent as well as reporter (fragment) ion exact mass produced by HCD.

**TMT reagent**	**Labeling mass change**	**HCD reporter ion exact mass**
TMT10plex-126	229.162932	126.127726
TMT10plex-127N	229.162932	127.124761
TMT10plex-127C	229.162932	127.131081
TMT10plex-128N	229.162932	128.128116
TMT10plex-128C	229.162932	128.134436
TMT10plex-129N	229.162932	129.131471
TMT10plex-129C	229.162932	129.137790
TMT10plex-130N	229.162932	130.134825
TMT10plex-130C	229.162932	130.141145
TMT10plex-131	229.162932	131.138180

Table S3. MS2 transition windows and precursors of the targeted tPRM method.

**Compound**	**Precursor (m/z)**	**Precursor isolation window (min)**
Aspartic acid	363.2077	9 - 16
IS Aspartic acid	368.2167	9 - 16
Threonine	349.2285	12.8 - 17.5
IS Threonine	354.2375	12.8 - 17.5
Alanine	319.2179	13.5 - 17.5
IS Alanine	323.2239	13.5 - 17.5
Glutamic acid	377.2234	13.9 - 18
IS Glutamic acid	383.2354	13.9 - 18
Serine	335.2128	14.5 - 18.5
IS Serine	339.2000	14.5 - 18.5
Proline	345.2335	16.9 - 21
IS Proline	351.2455	16.9 - 21
Lysine	303.2230	17 - 20
IS Lysine	308.2334	17 - 20
Cystine	350.1821	18.2 - 21
IS Cystine	354.1881	18.2 - 21
Valine	347.2492	19.8 - 23
IS Valine	353.2612	19.8 - 23
Methionine	379.2213	20 - 23.5
IS Methionine	385.2333	20 - 23.5
Tyrosine	411.2441	20.2 - 23
IS Tyrosine	421.2681	20.2 - 23
Isoleucine	361.2648	23.7 - 25
IS Isoleucine	368.2798	23.7 - 25
Leucine	361.2648	24.9 - 27
IS leucine	368.2798	24.9 - 27
Phenylalanine	395.2492	26 - 28.5
IS Phenylalanine	405.2732	26 - 28.5

Table S4. Exclusion mass list.

An exclusion mass list was produced based on background ions in both A-eluent, B-eluent and labeling byproduct *m/z* values with relative intensity above 1% in mass spectra.

Number	m/z		Number	m/z		Number	m/z		Number	m/z
1	202.0773		21	280.1618		41	145.1220		61	229.1403
2	149.0230		22	217.1066		42	520.1378		62	315.2132
3	279.1585		23	221.1531		43	229.0009		63	301.1401
4	445.1187		24	223.0631		44	163.1114		64	339.3440
5	219.1738		25	209.0595		45	256.2629		65	215.1247
6	429.0875		26	136.0213		46	268.9778		66	360.3226
7	355.0690		27	447.1159		47	220.1772		67	354.3176
8	203.0806		28	209.1531		48	277.1792		68	273.1664
9	371.1002		29	150.0263		49	667.1746		69	441.2961
10	271.2625		30	371.3146		50	238.0129		70	149.0229
11	203.0850		31	235.2051		51	372.0999			
12	235.1687		32	199.1689		52	163.0750			
13	519.1373		33	170.0961		53	593.1560			
14	220.1817		34	253.1792		54	357.0660			
15	446.1189		35	231.0337		55	151.1114			
16	503.1062		36	161.0957		56	202.0771			
17	391.2832		37	229.0181		57	203.0804			
18	205.0854		38	431.0845		58	338.3408			
19	299.0610		39	359.0275		59	203.0849			
20	430.0875		40	356.0686		60	271.1872			

Table S5. mzMine batch process.

Batch process steps in the mzMine for the untargeted metabolomics experiment results.

Step	Description	Settings
**Data Import**	Imports raw spectral data from files.	**File Names**: List of .mzML files
**Mass Detection (MS1)**	Detects masses in the MS1 spectral level.	**Mass Detector**: Factor of lowest signal; **Noise Factor**: 2; **Denormalize Fragment Scans**: Enabled
**Mass Detection (MS2)**	Detects masses in the MS2 spectral level.	**Mass Detector**: Factor of lowest signal; **Noise Factor**: 1; **Denormalize Fragment Scans**: Enabled
**Chromatogram Building (ADAP)**	Constructs chromatograms from the detected masses.	**Minimum Consecutive Scans**: 4; **Minimum Intensity**: 2000.0; **Minimum Absolute Height**: 10000; **m/z Tolerance**: 0.01 absolute, 10.0 ppm
**Smoothing**	Applies smoothing algorithms to chromatograms.	**Algorithm**: Savitzky-Golay; **Polynomial Order**: 3
**Local Minimum Resolver Deconvolution**	Resolves overlapping signals within chromatograms.	**Algorithm**: Local Minimum Resolver; **MS/MS Scan Pairing**: Enabled; **Dimension**: Retention Time; **Chromatographic Threshold**: 85%; **Minimum Search Range RT**: 0.3; **Minimum Relative Height**: 0; **Minimum Absolute Height**: 10000; **Min Ratio of Peak Top/Edge**: 1.8; **Peak Duration Range**: 0-9.01; **Minimum Scans**: 4
**13C Isotope Grouper**	Groups isotopic peaks together.	**m/z Tolerance**: 0.01 absolute, 3.0 ppm; **RT Tolerance**: 0.1 minute; **Monotonic Shape**: Enabled; **Maximum Charge**: 2; **Representative Isotope**: Most intense; **Never Remove Feature with MS2**: Enabled
**Isotopic Peak Finder**	Identifies specific isotopic peaks within the MS data.	**Chemical Elements**: H, C, N, O, S; **m/z Tolerance**: 0.01 absolute, 3.0 ppm; **Maximum Charge of Isotope m/z**: 1; **Search in Scans**: Single most intense
**Feature Alignment (RANSAC)**	Aligns features across different samples.	**m/z Tolerance**: 0.005 absolute, 5.0 ppm; **RT Tolerance**: 3.0 minutes; **RT Tolerance After Correction**: 0.2 minutes; **RANSAC Iterations**: 0 (not limited); **Minimum Number of Points**: 50%; **Threshold Value**: 0.1
**Feature Lists Rows Filter**	Filters the feature rows based on selections.	**Minimum Aligned Features (Samples)**: 1 or 0%; **Feature with MS2 Scans**: Enabled; **Never Remove Feature with MS2**: Enabled
**Peak Finder (Multithreaded)**	Identifies and fills gaps in the feature list.	**Intensity Tolerance**: 20%; **m/z Tolerance**: 0.01 absolute or 10 ppm; **Retention Time Tolerance**: 1.5 minutes; **Minimum Scans**: 1
**Duplicate Filtering**	Removes duplicate features.	**Filter Mode**: New average; **m/z Tolerance**: 0.01 absolute, 2.0 ppm; **RT Tolerance**: 0.3 minute
**Correlation Grouping (MetaCorrelate)**	Groups features based on correlation analysis.	**RT Tolerance**: 0.33 minutes; **Minimum Feature Height**: 0; **Intensity Threshold for Correlation**: 2; **Feature Shape Correlation**: Enabled; **Feature Height Correlation**: Enabled
**Ion Identity Networking**	Constructs networks of ion identities based on mass spectral data.	**m/z Tolerance**: 0.01 absolute or 3 ppm; **Min Height**: 0; **Ion Identity Library**: As suggested; **Annotation Refinement**: Enabled
**MSMS Spectral Networking**	Links MS/MS spectra across samples to build spectral networks.	**m/z Tolerance (MS2)**: 0.01; **Only Best MS2 Scan**: Enabled; **Max Precursor m/z Delta**: 500; **Minimum Matched Signals**: 4; **Minimum Cosine Similarity**: 0.7
**Data Export as CSV**	Exports the processed data to a CSV file for further analysis or reporting.	**Export All**: Enabled

Figure S1. Calibration Graphs.

Calibration graphs of partially validated (as in article Table 1) analyte quantitation.

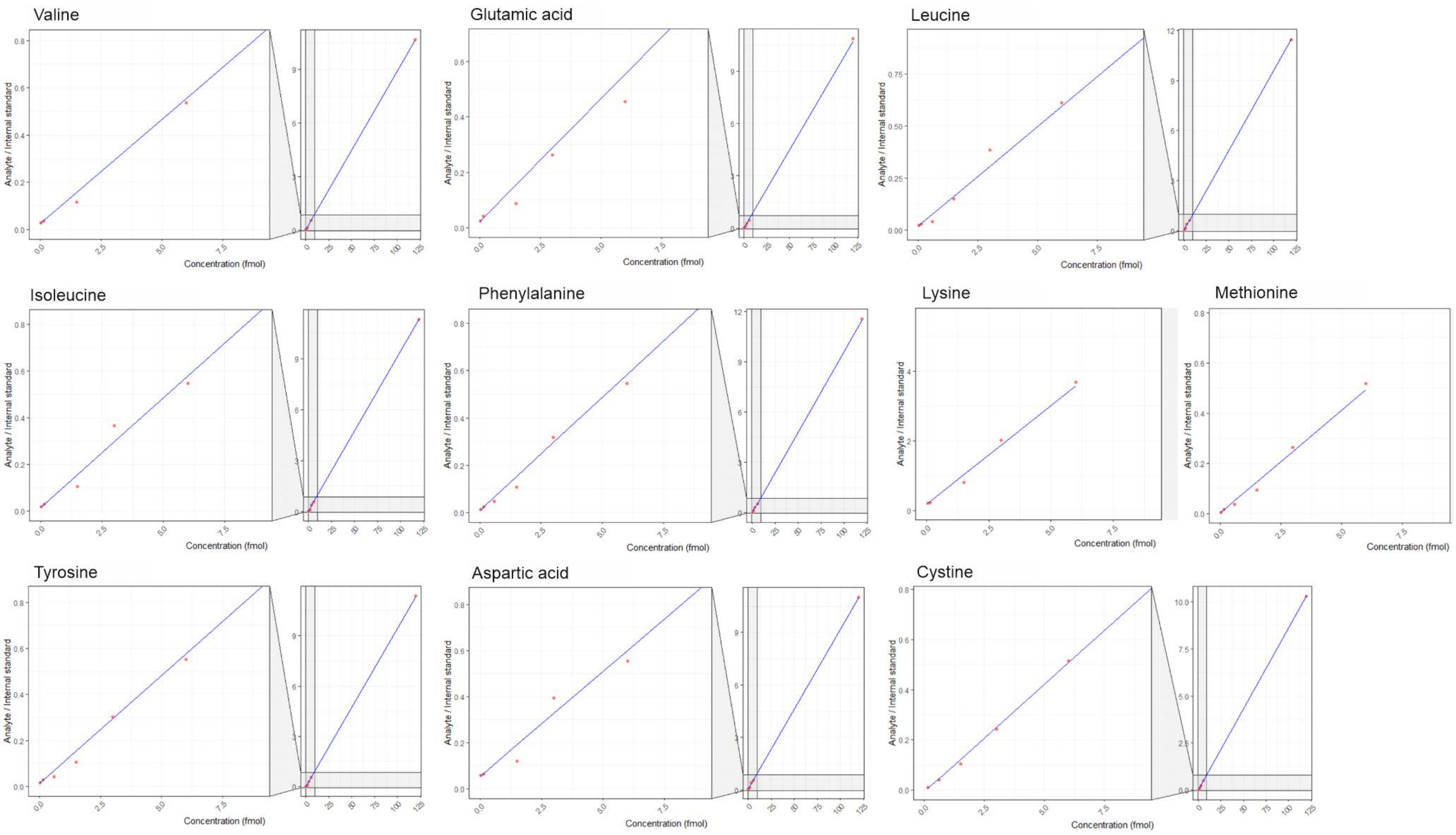

Figure S2. Retention time comparison on nLC and UHPLC.

Comparison of retention time of nLC and previously[2] performed UHPLC analysis of TMT labeled analytes. The analytes present but unquantifiable (glycine, serine, arginine and histidine) here are also analytes with the lowest retention time in the previous experiments suggesting their poor retention during the sample loading and chromatogram building steps of nLC operation.

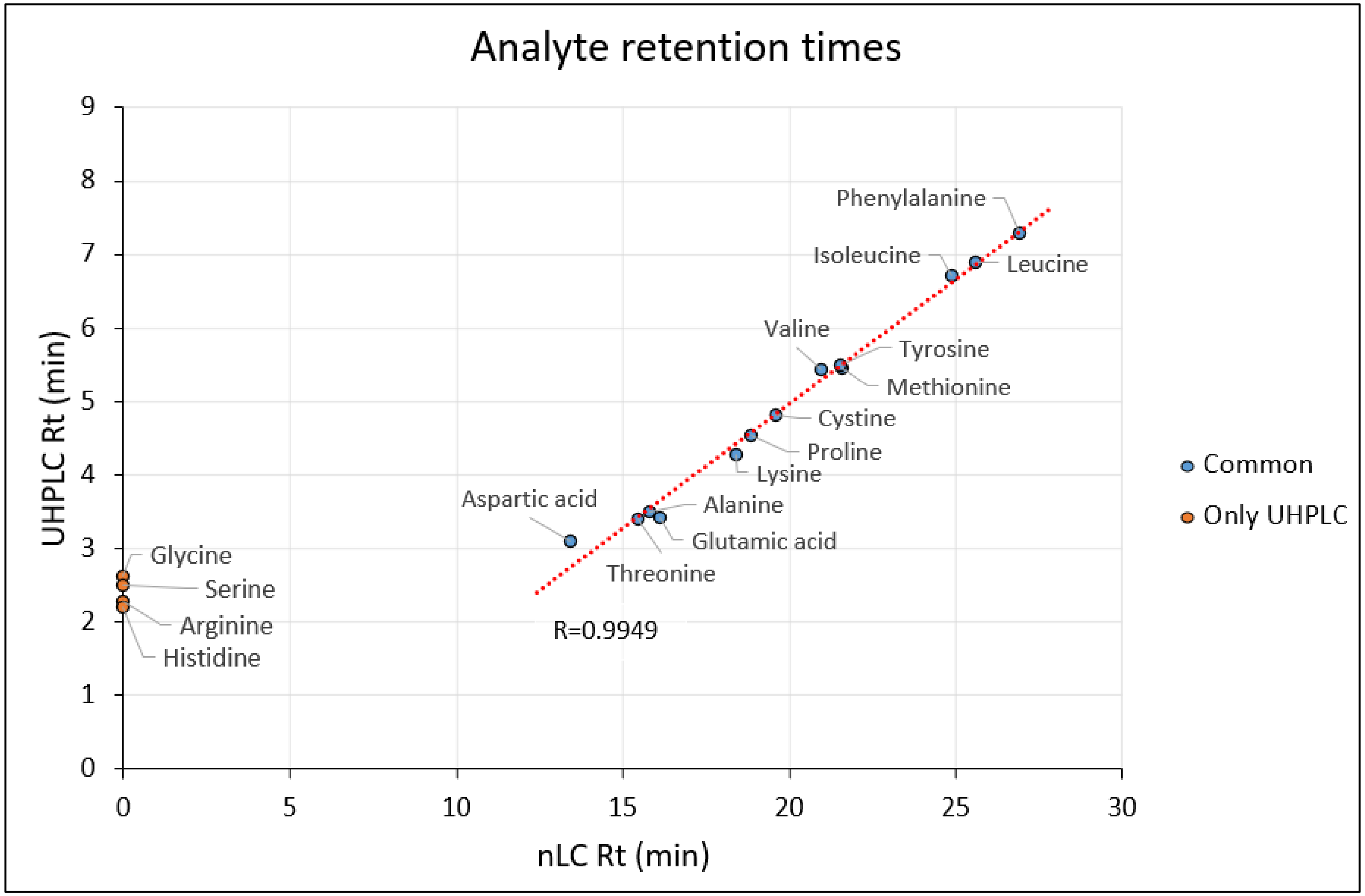

Figure S3. Heatmaps of single cells.

Heatmap of amine features attained using untargeted metabolomics approach on single cells. Intensities are intra-scan normalized to the carrier. From the 2178 features with cell specific reporter ions along with carrier signal, 249 were common in at least 25% of the samples (first zoom section). An exemplary section showing commonness and missingness of 21 features between replicates (second zoom section).

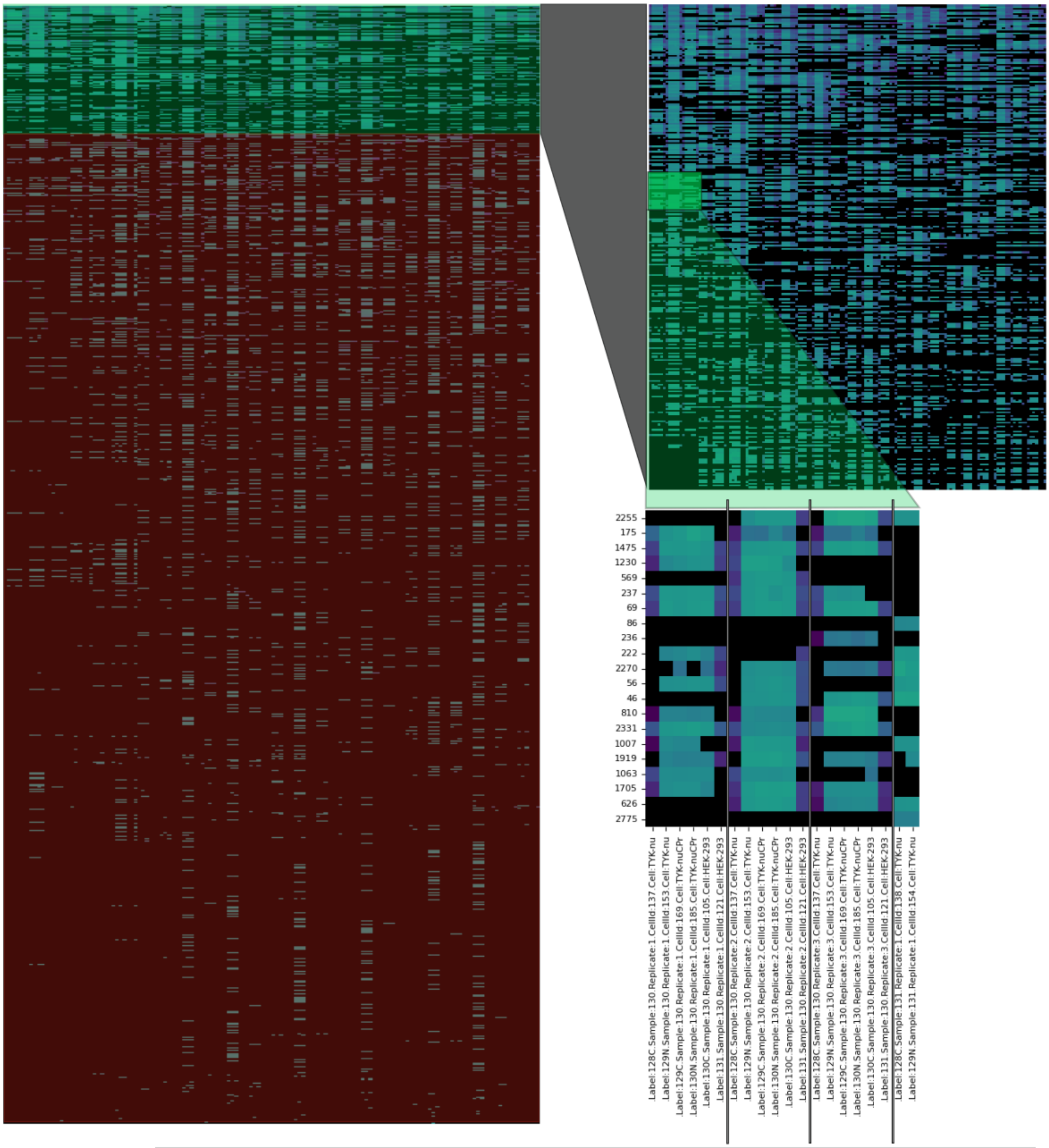

Figure S4. PCA of full data and RLS selection.

Principal components were computed and the first two principal components (PC1 and PC2) were then plotted for both the full dataset (A) and the top 100 features selected by Greedy Forward RLS (B).

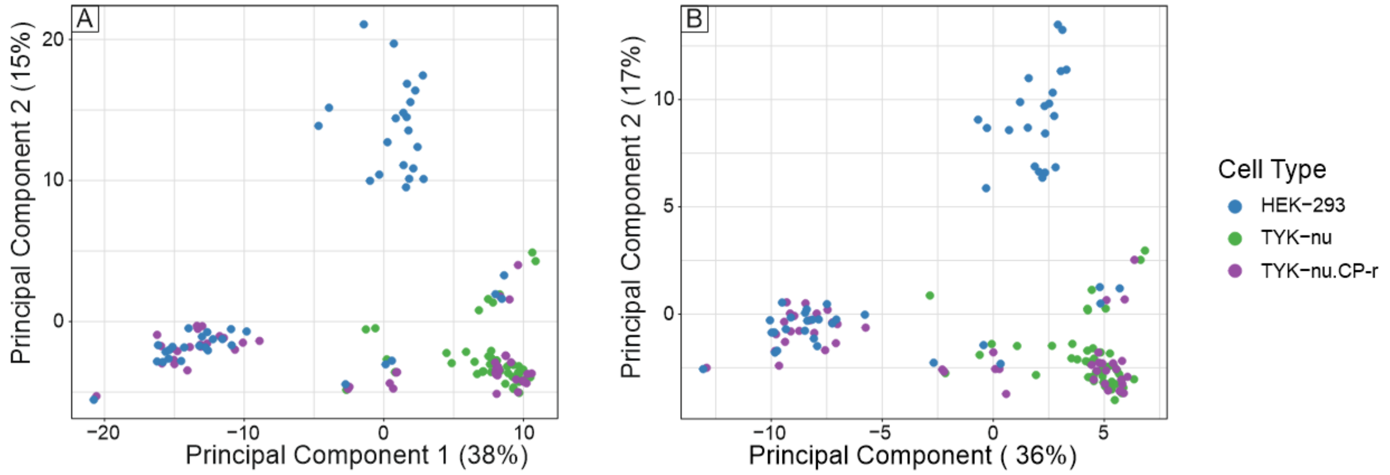

Figure S5. Example of MS2 scan matching for glutamic acid annotation.

Features were annotated using MS2 spectra against previously[2] measured TMT10plex labeled amines (A) and MS2 spectra of unlabeled analyte from GNPS2 database universal spectrum identifier (USI) interface[26] (GNPS Spectrum USI: mzspec_GNPS_GNPS-LIBRARY_accession_CCMSLIB00000081783) with added TMT10plex labeling mass +229.162932 (B). Matches were evaluated using Cosine similarity and proportion of TIC explained by the matching reference ions. To avoid artificially inflating the cosine similarity score with other TMT labeling products only (non-reporter ion) spectra above 132 Da and relative abundance above 0.1% were considered.

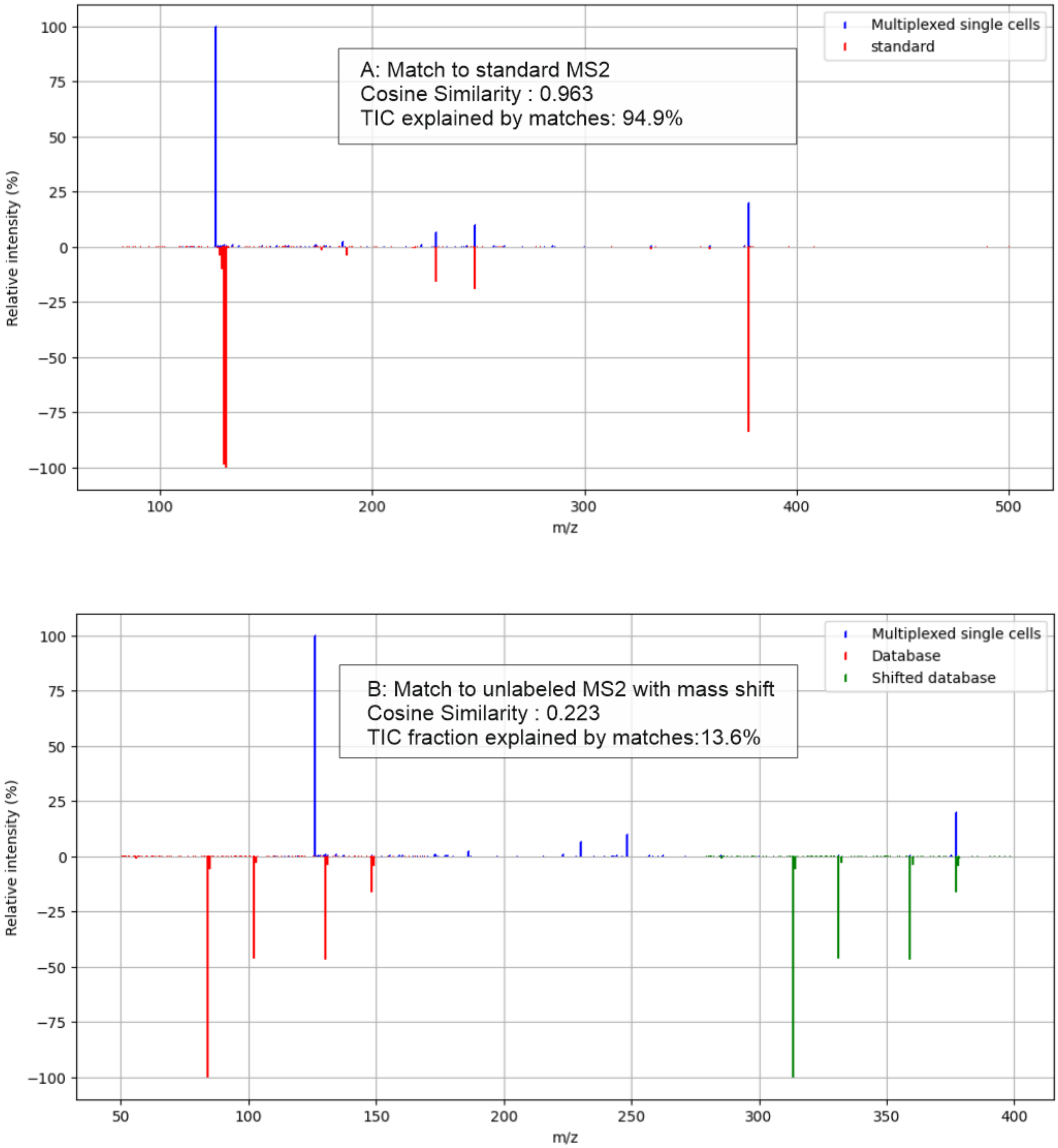

References

(1) Végvári, Á.; Rodriguez, J. E.; Zubarev, R. A. Single Cell Proteomics Using Multiplexed Isobaric Labeling for Mass Spectrometric Analysis. In *Single-Cell Protein Analysis*; 2022; pp 113–127. https://doi.org/10.1007/978-1-0716-1771-7_8.

(2) Heininen, J.; Julku, U.; Myöhänen, T.; Kotiaho, T.; Kostiainen, R. Multiplexed Analysis of Amino Acids in Mice Brain Microdialysis Samples Using Isobaric Labeling and Liquid Chromatography-High Resolution Tandem Mass Spectrometry. *J. Chromatogr. A* **2021**, *1656*. https://doi.org/10.1016/j.chroma.2021.462537.

(3) Leduc, A.; Huffman, R. G.; Cantlon, J.; Khan, S.; Slavov, N. Exploring Functional Protein Covariation across Single Cells Using NPOP. *Genome Biol.* **2022**, *23* (1), 1–31. https://doi.org/10.1186/s13059-022-02817-5.

(4) Adams, K. J.; Pratt, B.; Bose, N.; Dubois, L. G.; St. John-Williams, L.; Perrott, K. M.; Ky, K.; Kapahi, P.; Sharma, V.; MacCoss, M. J.; Moseley, M. A.; Colton, C. A.; MacLean, B. X.; Schilling, B.; Thompson, J. W. Skyline for Small Molecules: A Unifying Software Package for Quantitative Metabolomics. *J. Proteome Res.* **2020**, *19* (4), 1447–1458. https://doi.org/10.1021/acs.jproteome.9b00640.

(5) RStudio Team. RStudio: Integrated Development Environment for R. Boston, MA 2020.

(6) R Core Team. *R: A language and environment for statistical computing.* R Foundation for Statistical Computing, Vienna, Austria. /www.R-project.org/.

(7) Wickham, H.; Averick, M.; Bryan, J.; Chang, W.; McGowan, L.; François, R.; Grolemund, G.; Hayes, A.; Henry, L.; Hester, J.; Kuhn, M.; Pedersen, T.; Miller, E.; Bache, S.; Müller, K.; Ooms, J.; Robinson, D.; Seidel, D.; Spinu, V.; Takahashi, K.; Vaughan, D.; Wilke, C.; Woo, K.; Yutani, H. Welcome to the Tidyverse. *J. Open Source Softw.* **2019**, *4* (43), 1686. https://doi.org/10.21105/joss.01686.

(8) Gatto, L.; Gibb, S.; Rainer, J. MSnbase, Efficient and Elegant R-Based Processing and Visualization of Raw Mass Spectrometry Data. *J. Proteome Res.* **2021**, *20* (1), 1063–1069. https://doi.org/10.1021/acs.jproteome.0c00313.

(9) Pedersen, T. L. Ggforce: Accelerating "Ggplot2." 2024.

(10) Wickham, H.; Pedersen, T. L.; Seidel, D. Scales: Scale Functions for Visualization. 2023.

(11) Neuwirth, E. RColorBrewer: ColorBrewer Palettes. 2022.

(12) Group, I. E. W. Validation of Analytical Procedures: Text and Methodology Q2(R1). In *Encyclopedia of Toxicology: Third Edition*; 2004; Vol. 4, pp 1070–1072. https://doi.org/10.1016/B978-0-12-386454-3.00861-7.

(13) Chambers, M. C.; MacLean, B.; Burke, R.; Amodei, D.; Ruderman, D. L.; Neumann, S.; Gatto, L.; Fischer, B.; Pratt, B.; Egertson, J.; Hoff, K.; Kessner, D.; Tasman, N.; Shulman, N.; Frewen, B.; Baker, T. A.; Brusniak, M. Y.; Paulse, C.; Creasy, D.; Flashner, L.; Kani, K.; Moulding, C.; Seymour, S. L.; Nuwaysir, L. M.; Lefebvre, B.; Kuhlmann, F.; Roark, J.; Rainer, P.; Detlev, S.; Hemenway, T.; Huhmer, A.; Langridge, J.; Connolly, B.; Chadick, T.; Holly, K.; Eckels, J.; Deutsch, E. W.; Moritz, R. L.; Katz, J. E.; Agus, D. B.; MacCoss, M.; Tabb, D. L.; Mallick, P. A Cross-Platform Toolkit for Mass Spectrometry and Proteomics. *Nat. Biotechnol.* **2012**, *30* (10), 918–920. https://doi.org/10.1038/nbt.2377.

(14) Schmid, R.; Heuckeroth, S.; Korf, A.; Smirnov, A.; Myers, O.; Dyrlund, T. S.; Bushuiev, R.; Murray, K. J.; Hoffmann, N.; Lu, M.; Sarvepalli, A.; Zhang, Z.; Fleischauer, M.; Dührkop, K.; Wesner, M.; Hoogstra, S. J.; Rudt, E.; Mokshyna, O.; Brungs, C.; Ponomarov, K.; Mutabdžija, L.; Damiani, T.; Pudney, C. J.; Earll, M.; Helmer, P. O.; Fallon, T. R.; Schulze, T.; Rivas-Ubach, A.; Bilbao, A.; Richter, H.; Nothias, L. F.; Wang, M.; Orešič, M.; Weng, J. K.; Böcker, S.; Jeibmann, A.; Hayen, H.; Karst, U.;

Dorrestein, P. C.; Petras, D.; Du, X.; Pluskal, T. Integrative Analysis of Multimodal Mass Spectrometry Data in MZmine 3. *Nat. Biotechnol.* **2023**, *41* (4), 447–449. https://doi.org/10.1038/s41587-023-01690-2.

(15) Van Der Walt, S.; Colbert, S. C.; Varoquaux, G. The NumPy Array: A Structure for Efficient Numerical Computation. *Comput. Sci. Eng.* **2011**, *13* (2), 22–30. https://doi.org/10.1109/MCSE.2011.37.

(16) Virtanen, P.; Gommers, R.; Oliphant, T. E.; Haberland, M.; Reddy, T.; Cournapeau, D.; Burovski, E.; Peterson, P.; Weckesser, W.; Bright, J.; van der Walt, S. J.; Brett, M.; Wilson, J.; Millman, K. J.; Mayorov, N.; Nelson, A. R. J.; Jones, E.; Kern, R.; Larson, E.; Carey, C. J.; Polat, İ.; Feng, Y.; Moore, E. W.; VanderPlas, J.; Laxalde, D.; Perktold, J.; Cimrman, R.; Henriksen, I.; Quintero, E. A.; Harris, C. R.; Archibald, A. M.; Ribeiro, A. H.; Pedregosa, F.; van Mulbregt, P.; Vijaykumar, A.; Bardelli, A. Pietro; Rothberg, A.; Hilboll, A.; Kloeckner, A.; Scopatz, A.; Lee, A.; Rokem, A.; Woods, C. N.; Fulton, C.; Masson, C.; Häggström, C.; Fitzgerald, C.; Nicholson, D. A.; Hagen, D. R.; Pasechnik, D. V.; Olivetti, E.; Martin, E.; Wieser, E.; Silva, F.; Lenders, F.; Wilhelm, F.; Young, G.; Price, G. A.; Ingold, G. L.; Allen, G. E.; Lee, G. R.; Audren, H.; Probst, I.; Dietrich, J. P.; Silterra, J.; Webber, J. T.; Slavič, J.; Nothman, J.; Buchner, J.; Kulick, J.; Schönberger, J. L.; de Miranda Cardoso, J. V.; Reimer, J.; Harrington, J.; Rodríguez, J. L. C.; Nunez-Iglesias, J.; Kuczynski, J.; Tritz, K.; Thoma, M.; Newville, M.; Kümmerer, M.; Bolingbroke, M.; Tartre, M.; Pak, M.; Smith, N. J.; Nowaczyk, N.; Shebanov, N.; Pavlyk, O.; Brodtkorb, P. A.; Lee, P.; McGibbon, R. T.; Feldbauer, R.; Lewis, S.; Tygier, S.; Sievert, S.; Vigna, S.; Peterson, S.; More, S.; Pudlik, T.; Oshima, T.; Pingel, T. J.; Robitaille, T. P.; Spura, T.; Jones, T. R.; Cera, T.; Leslie, T.; Zito, T.; Krauss, T.; Upadhyay, U.; Halchenko, Y. O.; Vázquez-Baeza, Y. SciPy 1.0: Fundamental Algorithms for Scientific Computing in Python. *Nat. Methods* **2020**, *17* (3), 261–272. https://doi.org/10.1038/s41592-019-0686-2.

(17) Pedregosa, F.; Varoquaux, G.; Gramfort, A.; Michel, V.; Thirion, B.; Grisel, O.; Blondel, M.; Prettenhofer, P.; Weiss, R.; Dubourg, V.; Vanderplas, J.; Passos, A.; Cournapeau, D.; Brucher, M.; Perrot, M.; Duchesnay, E. Scikit-Learn: Machine Learning in {P}ython. *J. Mach. Learn. Res.* **2011**, *12*, 2825–2830.

(18) Behdenna, A.; Colange, M.; Haziza, J.; Gema, A.; Appé, G.; Azencott, C. A.; Nordor, A. PyComBat, a Python Tool for Batch Effects Correction in High-Throughput Molecular Data Using Empirical Bayes Methods. *BMC Bioinformatics* **2023**, *24* (1), 1–9. https://doi.org/10.1186/s12859-023-05578-5.

(19) Johnson, W. E.; Li, C.; Rabinovic, A. Adjusting Batch Effects in Microarray Expression Data Using Empirical Bayes Methods. *Biostatistics* **2007**, *8* (1), 118–127. https://doi.org/10.1093/biostatistics/kxj037.

(20) Hunter, J. D. Matplotlib: A 2D Graphics Environment. *Comput. Sci. Eng.* **2007**, *9* (3), 90–95. https://doi.org/10.1109/MCSE.2007.55.

(21) Waskom, M. Seaborn: Statistical Data Visualization. *J. Open Source Softw.* **2021**, *6* (60), 3021. https://doi.org/10.21105/joss.03021.

(22) Pahikkala, T.; Airola, A. RLscore: Regularized Least-Squares Learners. *J. Mach. Learn. Res.* **2016**, *17*, 1–5.

(23) Wishart, D. S.; Guo, A. C.; Oler, E.; Wang, F.; Anjum, A.; Peters, H.; Dizon, R.; Sayeeda, Z.; Tian, S.; Lee, B. L.; Berjanskii, M.; Mah, R.; Yamamoto, M.; Jovel, J.; Torres-Calzada, C.; Hiebert-Giesbrecht, M.; Lui, V. W.; Varshavi, D.; Varshavi, D.; Allen, D.; Arndt, D.; Khetarpal, N.; Sivakumaran, A.; Harford, K.; Sanford, S.; Yee, K.; Cao, X.; Budinski, Z.; Liigand, J.; Zhang, L.; Zheng, J.; Mandal, R.; Karu, N.; Dambrova, M.; Schiöth, H. B.; Greiner, R.; Gautam, V. HMDB 5.0: The Human Metabolome Database for 2022. *Nucleic Acids Res.* **2022**, *50* (D1), D622–D631. https://doi.org/10.1093/nar/gkab1062.

(24) Wang, M.; Carver, J. J.; Phelan, V. V.; Sanchez, L. M.; Garg, N.; Peng, Y.; Nguyen, D. D.; Watrous, J.; Kapono, C. A.; Luzzatto-Knaan, T.; Porto, C.; Bouslimani, A.; Melnik, A. V.; Meehan, M. J.; Liu, W. T.; Crüsemann, M.; Boudreau, P. D.; Esquenazi, E.; Sandoval-Calderón, M.; Kersten, R. D.; Pace,

L. A.; Quinn, R. A.; Duncan, K. R.; Hsu, C. C.; Floros, D. J.; Gavilan, R. G.; Kleigrewe, K.; Northen, T.; Dutton, R. J.; Parrot, D.; Carlson, E. E.; Aigle, B.; Michelsen, C. F.; Jelsbak, L.; Sohlenkamp, C.; Pevzner, P.; Edlund, A.; McLean, J.; Piel, J.; Murphy, B. T.; Gerwick, L.; Liaw, C. C.; Yang, Y. L.; Humpf, H. U.; Maansson, M.; Keyzers, R. A.; Sims, A. C.; Johnson, A. R.; Sidebottom, A. M.; Sedio, B. E.; Klitgaard, A.; Larson, C. B.; Boya, C. A. P.; Torres-Mendoza, D.; Gonzalez, D. J.; Silva, D. B.; Marques, L. M.; Demarque, D. P.; Pociute, E.; O'Neill, E. C.; Briand, E.; Helfrich, E. J. N.; Granatosky, E. A.; Glukhov, E.; Ryffel, F.; Houson, H.; Mohimani, H.; Kharbush, J. J.; Zeng, Y.; Vorholt, J. A.; Kurita, K. L.; Charusanti, P.; McPhail, K. L.; Nielsen, K. F.; Vuong, L.; Elfeki, M.; Traxler, M. F.; Engene, N.; Koyama, N.; Vining, O. B.; Baric, R.; Silva, R. R.; Mascuch, S. J.; Tomasi, S.; Jenkins, S.; Macherla, V.; Hoffman, T.; Agarwal, V.; Williams, P. G.; Dai, J.; Neupane, R.; Gurr, J.; Rodríguez, A. M. C.; Lamsa, A.; Zhang, C.; Dorrestein, K.; Duggan, B. M.; Almaliti, J.; Allard, P. M.; Phapale, P.; Nothias, L. F.; Alexandrov, T.; Litaudon, M.; Wolfender, J. L.; Kyle, J. E.; Metz, T. O.; Peryea, T.; Nguyen, D. T.; VanLeer, D.; Shinn, P.; Jadhav, A.; Müller, R.; Waters, K. M.; Shi, W.; Liu, X.; Zhang, L.; Knight, R.; Jensen, P. R.; Palsson, B.; Pogliano, K.; Linington, R. G.; Gutiérrez, M.; Lopes, N. P.; Gerwick, W. H.; Moore, B. S.; Dorrestein, P. C.; Bandeira, N. Sharing and Community Curation of Mass Spectrometry Data with Global Natural Products Social Molecular Networking. *Nat. Biotechnol.* **2016**, *34* (8), 828–837. https://doi.org/10.1038/nbt.3597.

(25) Schymanski, E. L.; Jeon, J.; Gulde, R.; Fenner, K.; Ruff, M.; Singer, H. P.; Hollender, J. Identifying Small Molecules via High Resolution Mass Spectrometry: Communicating Confidence. *Environ. Sci. Technol.* **2014**, *48* (4), 2097–2098. https://doi.org/10.1021/es5002105.

(26) Bittremieux, W.; Chen, C.; Dorrestein, P. C.; Schymanski, E. L.; Schulze, T.; Neumann, S.; Meier, R.; Rogers, S.; Wang, M. Universal MS/MS Visualization and Retrieval with the Metabolomics Spectrum Resolver Web Service. *bioRxiv* **2020**, 2020.05.09.086066. https://doi.org/10.1101/2020.05.09.086066.
